# Brain Metabolism During A Lower Extremity Voluntary Movement Task in Children With Spastic Cerebral Palsy

**DOI:** 10.3389/fnhum.2020.00159

**Published:** 2020-05-25

**Authors:** Eileen G. Fowler, William L. Oppenheim, Marcia B. Greenberg, Loretta A. Staudt, Shantanu H. Joshi, Daniel H. S. Silverman

**Affiliations:** ^1^Center for Cerebral Palsy, Department of Orthopaedic Surgery, University of California, Los Angeles, Los Angeles, CA, United States; ^2^Tarjan Center at UCLA, Los Angeles, CA, United States; ^3^Department of Neurology, University of California, Los Angeles, Los Angeles, CA, United States; ^4^Department of Bioengineering, University of California, Los Angeles, Los Angeles, CA, United States; ^5^Department of Molecular and Medical Pharmacology, University of California, Los Angeles, Los Angeles, CA, United States; ^6^Ahmanson Translational Imaging Division, UCLA Health System, Los Angeles, CA, United States

**Keywords:** spastic cerebral palsy, PET—positron emission tomography, brain metabolism, selective voluntary motor control, ankle motor task

## Abstract

Reduced selective voluntary motor control (SVMC) is a primary impairment due to corticospinal tract (CST) injury in spastic cerebral palsy (CP). There are few studies of brain metabolism in CP and none have examined brain metabolism during a motor task. Nine children with bilateral spastic CP [Age: 6-11 years, Gross Motor Function Classification System (GMFCS) Levels II–V] completed this study. SVMC was evaluated using Selective Control Assessment of the Lower Extremity (SCALE) ranging from 0 (absent) to 10 (normal). Brain metabolism was measured using positron emission tomography (PET) scanning in association with a selective ankle motor task. Whole brain activation maps as well as ROI averaged metabolic activity were correlated with SCALE scores. The contralateral sensorimotor and superior parietal cortex were positively correlated with SCALE scores (*p* < 0.0005). In contrast, a negative correlation of metabolic activity with SCALE was found in the cerebellum (*p* < 0.0005). Subsequent ROI analysis showed that both ipsilateral and contralateral cerebellar metabolism correlated with SCALE but the relationship for the ipsilateral cerebellum was stronger (*R*^2^ = 0.80, *p* < 0.001 vs. *R*^2^ = 0.46, *p* = 0.045). Decreased cortical and increased cerebellar activation in children with less SVMC may be related to task difficulty, activation of new motor learning paradigms in the cerebellum and potential engagement of alternative motor systems when CSTs are focally damaged. These results support SCALE as a clinical correlate of neurological impairment.

## Introduction

Children with spastic cerebral palsy (CP) have developmental brain injuries primarily affecting the motor systems. Impairments of motor control are observed early in development (Fetters et al., 2004; Sargent et al., 2017) often preceding the detection of spasticity in children with CP. Deficits in gross motor function including mobility, strength, and balance are additional impairments. Spastic CP results from damage to the periventricular white matter containing descending motor tracts including the corticospinal tracts (CSTs) responsible for voluntary motor control (Bax et al., 2006; Volpe, 2009). White matter damage including the CSTs has been described and quantified in CP and correlated with motor and sensory function measures using magnetic resonance imaging (MRI) with diffusion tensor imaging (DTI) techniques (Hoon et al., 2009; Lee et al., 2011). While damage to the developing CSTs is a primary etiology in spastic CP, resulting compensatory adaptations have not been adequately studied relative to brain structure and activity, especially for lower extremity function in patients with bilateral involvement.

CSTs that originate in the motor cortex are responsible for skilled voluntary movement or selective motor control. The term “selective voluntary motor control” (SVMC) indicates the deliberate performance of isolated movements upon request (Fowler et al., 2010). Children with spastic CP and impaired SVMC may exhibit reduced speed of movement, mirror movements or abnormal reciprocal muscle activation patterns. Also, they are often unable to move their hip, knee and ankle joints in isolation, relying instead on closely coupled flexion and extension patterns to varying degrees (Fowler and Goldberg, 2009). In two studies, SVMC was more predictive of motor function than other aspects of CP (Østensjø et al., 2004; Voorman et al., 2007). Clinical measures of SVMC have been shown to correlate with mobility level (Fowler et al., 2009), gross motor function (Balzer et al., 2016; Noble et al., 2019) and gait (Fowler and Goldberg, 2009; Steele et al., 2015; Rha et al., 2016; Chruscikowski et al., 2017; Zhou et al., 2019).

Following a perinatal injury to the CSTs, alternative motor pathways develop that are forms of adaptive or maladaptive plasticity (Eyre, 2007; Friel et al., 2013; Gordon, 2016). This has been shown for animal models and the upper extremity of children with spastic hemiplegic CP. Ipsilateral CSTs from the uninvolved hemisphere can be preserved causing mirror movements and other impairments. In adults post stroke, it has been suggested that compensations by areas of the brain such as the rubrospinal tracts are utilized when damage to CSTs occur, producing synergistic flexor and extensor patterns in the involved extremities during voluntary movement (Yeo and Jang, 2010). While children with spastic CP sustain an early injury to the brain before the development of motor skills, similar compensatory pathways may be involved (Cahill-Rowley and Rose, 2014).

The structure of white matter tracts has been the focus of brain imaging studies in spastic CP (Scheck et al., 2012; Mailleux et al., 2020). Far less is known about neuromotor recruitment. Studies of brain activity during movement in CP have primarily focused on children with unilateral involvement during upper extremity fine motor or sensory tasks using functional magnetic resonance imaging (fMRI; Dinomais et al., 2013; Van de Winckel et al., 2013a,b). Only two fMRI studies investigating lower extremity movement in CP could be found (Phillips et al., 2007; Hilderley et al., 2018). All children were high functioning as they were able to walk and run independently (GMFCS level I) and could actively dorsiflex the ankle, which indicates a high level of selective movement. Despite these stringent inclusion criteria, excessive head movement during the motor task was problematic during fMRI data collection resulting in unusable data for some participants (Phillips et al., 2007) or limiting the number of available trials for analysis (Hilderley et al., 2018). Brain activation can also be studied using positron emission tomography (PET), a metabolic imaging technique that uses radioactive compounds to label functional brain metabolism (Phelps, 2000). A common biologically active molecule used is FDG, an analog of glucose. Regional glucose metabolism and accumulation represent the metabolic activity of the tissues (Alauddin, 2012). Radiotracer concentrations in specific regions of the brain are mapped on three-dimensional images of the brain that are reconstructed from MRI (Lee et al., 2007; Penny et al., 2011). Only one PET scan study could be found that investigated brain metabolism in CP (Lee et al., 2007) but it did not involve a motor task. An advantage of PET for evaluating neuromotor control is that motor task performance and tracer uptake occur before the imaging session. In contrast, the motor task occurs during imaging for fMRI requiring that head position be maintained during limb movement, excluding more children with CP from participation.

The purpose of this study was to examine the relationship between brain metabolism and SVMC in children with spastic bilateral CP during movement using PET. SVMC was evaluated using the Selective Control Assessment of the Lower Extremity (SCALE; Fowler et al., 2009). We hypothesized that a significant positive relationship between SCALE and the sensorimotor cortex would be found. Our secondary hypothesis was that significant correlations between SCALE and activation of other motor regions of the brain would be identified.

## Materials and Methods

### Participants

Children with spastic bilateral CP were recruited from clinics and the community *via* mailings and flyers. This study was approved by the Human Subject Committee at the University of California, Los Angeles, CA, USA. Informed assents and consents were obtained from the participants and their guardians. Participants were recruited from the Center for CP at UCLA/OIC as well as the surrounding Los Angeles community. Data used for this analysis were collected as baseline information for a larger treatment intervention study. Inclusion criteria were: (1) age between 4 and 12 years; (2) diagnosis of spastic form of CP; and (3) ability to remain still for a minimum of 15 min. Exclusion criteria were: (1) attention deficit or hyperactivity disorder; (2) seizure within the last 6 months; (3) participation in a research study that involves the use of radiation in the past 12 months; (4) fear of enclosed spaces, breathing or swallowing problems, dizziness, or fainting spells; (5) pacemaker, intrathecal baclofen pump or metal implants in the head or neck, other than tooth fillings; and (6) mechanical, cystic or other structural abnormalities on MRI. Following informed consent, each participant received a structural MRI. Participants who were unable to lie still or who had MRI exclusion factors were discontinued from study participation. A physical therapist assessed mobility using the Gross Motor Function Classification System (GMFCS; Palisano et al., 1997) and gross motor function using the Gross Motor Function Measure (GMFM; Bjornson et al., 1998).

### Selective Voluntary Motor Control Assessment

Before PET scanning, SVMC was assessed by a physical therapist using SCALE, a validated and reliable clinical tool, which was developed for individuals with spastic CP (Fowler et al., 2009; Balzer et al., 2016). This assessment incorporates components of CST function including selectivity, reciprocation, and speed as well as the presence of involuntary movement at other joints including mirror movements of the contralateral extremity. Hip, knee, ankle, subtalar and toe joints are assessed using an isolated, reciprocal movement pattern and each joint is scored 0 (Unable), 1 (Impaired) or 2 (Normal). Scores are summed resulting in a possible score from 0 (absent SVMC) to 10 (normal SVMC) for each lower limb.

### PET Scan Data Acquisition

PET scanning was used to acquire regional cerebral metabolic data. Participants were asked to fast for 3 h before the procedure to optimize glucose uptake. The process of injection and PET scan required approximately 2 h for completion. An intravenous line was placed and 185 MBq (FDG) was administered using standard aseptic technique. A single small blood sample was obtained to establish the starting level of blood glucose. PET scans were performed 40 min post-FDG injection.

During this uptake period, the participant performed an ankle movement task with the lower extremity that demonstrated the least impairment. The task was limited to one lower extremity to examine regions of the brain that controlled movement for the ipsilateral vs. contralateral limb. The child was instructed by a physical therapist to perform a series of isolated ankle dorsiflexion and plantar flexion movements that were initiated every 2 min followed by a brief rest period. If movement occurred at other joints, verbal feedback was provided for initial attempts but the feedback was discontinued if the motion was obligatory.

### Whole Brain Analysis

Relative quantification of regional brain activity was performed using NeuroQ™ (Syntermed Inc., Atlanta). This software corrects for tissue-based attenuation and then implements an algorithm for automatically measuring the number of radioactive events emitted by a positron source (gamma-ray lines of coincidence) per second detected by PET-scanner, emanating from pixel locations assigned by a computerized reconstruction algorithm. Statistical parametric mapping (SPM) methods were performed (Friston et al., 1995a,b) to co-register participant images and to reorient them into a standardized coordinate system using the SPM software package (Ashburner, 2009) from the Wellcome Department of Cognitive Neurology, Functional Imaging Laboratory (London, UK). Data were spatially smoothed and normalized to mean global activity as previously described (Silverman et al., 2011), except for a 12 mm (full-width half-maximum) smoothing filter that was applied to the images before statistical analysis. The set of pooled data were assessed with the t-statistic on a voxel-by-voxel basis, to identify the profile of voxels that significantly covaried with parameters characterizing each participant. To identify the anatomical label of the underlying voxel, we defined 240 standardized ROIs (sROIs) following the transformation of each PET scan to a template space (Tai et al., 1997) throughout the transaxial planes across the field of view. Normalized uptake values were determined for specific regions of the brain. Whole-brain voxel-wise Pearson correlations of metabolic activity vs. SCALE scores were performed. These results were correlated for multiple comparisons using cluster wise thresholding in SPM. All 240 sROIs and 47 volumes of interest (sVOIs) were used solely to obtain an anatomic parcellation of the brain and to identify the anatomical regions where there is cluster-wise statistical significance.

### Secondary ROI Analysis

In the case of bilateral activation, it was necessary to further identify whether the FDG uptake was ipsilateral or contralateral to the moving limb. Therefore, a secondary ROI analysis was performed exclusively for those regions. Mean voxel activity for the bilateral ROIs was calculated following the transformation of each PET scan to a template space. This value was automatically normalized to the mean activity measured throughout that brain scan for each ROI and was used to correlate with SCALE. Importantly, for correlation analysis, only the anatomical ROI-based average instead of the statistical ROI-based average was used to avoid circular analysis.

## Results

Participant characteristics are shown for 10 children who were enrolled and underwent baseline testing in Table 1. The average age was 9 years, 4 months. Motor impairment ranged from mild (GMFCS Level II, GMFM 72.2, SCALE 7 bilaterally) to severe (GMFCS Level V, GMFM 36.0, SCALE scores ≤ 3). Two participants exhibited bilateral SCALE score = 0. Four children could walk and six used wheelchairs as their primary mode of mobility. One participant was dropped from the study after enrollment due to a significant structural abnormality that was identified on MRI. Of the remaining nine participants, eight had white matter damage including periventricular leukomalacia on MRI. Bilateral volume loss of the thalamus was additionally reported for one of these children.

**Table 1:** Participant characteristics.

Demographics		*n* = 10
Age	Mean Age (SD) year, month	9, 4 (1, 4)
	Age range years	6–11
Gender	Male	6
	Female	4
Ethnicity	Hispanic	4
Race	African American	1
	Caucasian	9
GMFCS	II	2
	III	2
	IV	3
	V	3
CP Diagnosis-distribution	Diplegia	5
	Quadriplegia	4
	Total body involvement	1
GMFM	Mean (SD)	53.2 (11.9)
SCALE	Left Mean (SD)	2.7 (2.5)
	Right Mean (SD)	2.5 (2.6)

*SD, standard deviation; GMFCS, gross motor function classification system; GMFM, gross motor function measure; SCALE, selective control assessment of the lower extremity*.

During the ankle motor task, obligatory movement at other joints was observed in most participants. All six participants with low limb SCALE scores (0–2) were unable to isolate ankle motion and exhibited simultaneous hip and knee synergistic movement. The remaining three participants with limb scores of ≥5 exhibited impaired SVMC at their ankle, due to an inability to perform at least 15° of isolated ankle motion or the presence of movement at another joint. Mirror movement at the ankle was observed for one participant.

### Qualitative Visualization of Whole Brain Metabolic Activity

**Figure 1:**
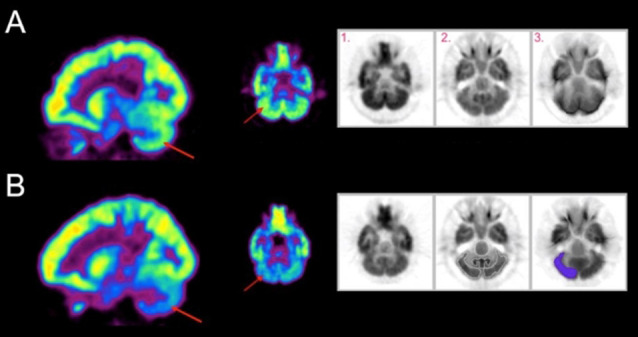
Metabolic activity maps are shown for **(A)** an individual with a low Selective Control Assessment of the Lower Extremity (SCALE) score and **(B)** an individual with a high SCALE score. Red arrows indicate the right cerebellum. The gray scale images (inset) for each row show 1. Participant’s original positron emission tomography (PET) scan, 2. Template PET scan on which standardized regions of interest are defined and 3. Activity resampled on the template. Relative hypoactivity was found in the cerebellum of the individual with a high SCALE score, indicated by the indigo color using a rainbow scale (violet being the lowest and red being the highest).

Exemplar metabolic activity maps for individuals with contrasting SCALE scores can be seen in Figure 1. The metabolic maps in the first two columns are color-coded (rainbow scale) showing normalized activity for sagittal and axial slices. A contrast between cerebellar activation levels for two participants with low vs. high SCALE scores can be seen.

### Whole-Brain Analysis

**Figure 2:**
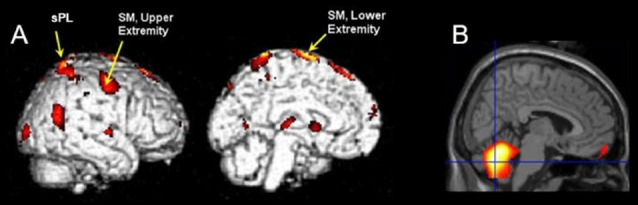
**(A)** A three-dimensional volumetric rendering of significant positive correlations between SCALE and metabolic activity in the sensorimotor and the superior parietal cortex during movement of the contralateral limb shown on the lateral view and medial cross-section overlaid on an atlas (*p* < 0.0005, cluster corrected). **(B)** Voxels show significant negative correlations of metabolic activity with SCALE in the cerebellum (*p* < 0.01 cluster corrected) on the medial slice. Color indicates a significant relationship and yellow indicates a stronger relationship as compared to red. sPL, superior parietal lobe, SM, sensorimotor cortex.

The results of SPM analyses examining correlations between metabolic activity and SCALE scores are shown in Figure 2. The sensorimotor (SM) and superior parietal (sPL) cortex contralateral to the moving limb were significantly positively correlated with SCALE scores (SM: *t* = 8.06, sPL: *t* = 6.70; *p* < 0.0005). In contrast, a significant negative correlation of metabolic activity with SCALE was found in the entire cerebellum (peak *t* = 7.23, *p* < 0.0005). As both sides of the cerebellum were correlated with SCALE, the level of activation for the side ipsilateral vs. contralateral to the moving limb was not apparent from the whole-brain analysis. Therefore, a secondary analysis at the ROI level was performed.

### ROI Analysis

**Figure 3:**
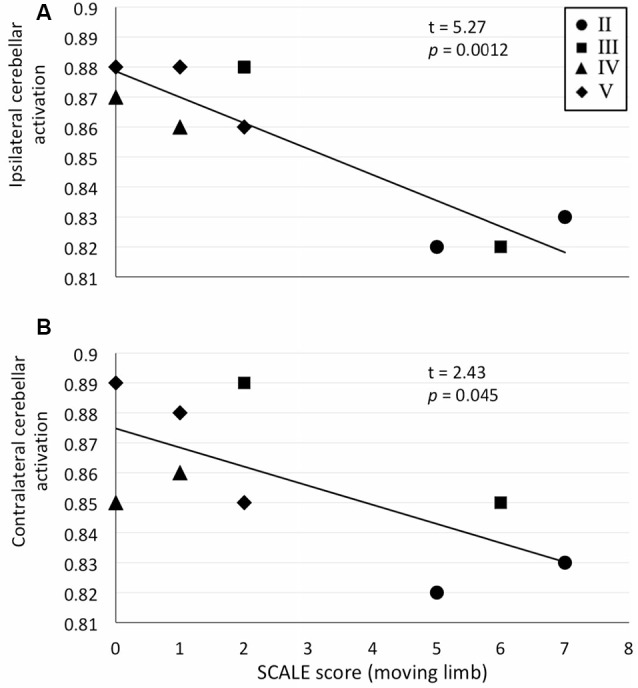
Correlation plots for ROI-averaged cerebellar activation (normalized units) vs. SCALE score (from 0 = absent to 10 = normal) for the moving limb. **(A)** Ipsilateral cerebellum vs. SCALE score, *R*^2^ = 0.80 and **(B)** Contralateral cerebellum vs. SCALE score, *R*^2^ = 0.46. Gross Motor Function Classification System (GMFCS) level for each participant are indicated as II–IV.

We exclusively selected the cerebellum for further ROI analysis as it showed bilateral activation in the whole-brain analysis. Significant correlations between cerebellar ROI-averaged activity and SCALE score for the moving limb were found. As justified by tests of linearity, parametric Pearson correlation coefficients were used. A strong significant correlation was found between the SCALE scores for the moving limb and the ipsilateral cerebellum (*t* = 5.27, *p* < 0.001, Figure 3A). While the correlation between the activation level of the contralateral cerebellum and SCALE score was significant, the relationship was not as strong (*t* = 2.43, *p* = 0.045, Figure 3B).

## Discussion

This is the first study to document a relationship between impaired lower extremity SVMC and brain metabolic activity in children with CP. Previous researchers reported greater metabolism in bilateral motor and visual cortices and the cerebellum in children with spastic CP relative to typically developing children using PET; however, a motor task was not performed (Lee et al., 2007). As expected, children with higher levels of motor control in the present study exhibited greater activity in the cortical areas associated with motor function (sensorimotor and superior parietal cortices) contralateral to the moving limb. This finding is consistent with the primary motor cortex being the largest source of CSTs and the sensory cortex providing feedback during motor tasks. Further, the superior parietal cortex is known to be involved in adjusting posture and guiding movement of the limbs, particularly concerning visual-spatial perception and body awareness (Wolpert et al., 1998; Wolbers et al., 2003). A related finding from a fMRI study has shown activations in the primary motor and sensory areas in healthy adults performing a similar ankle dorsiflexion and plantarflexion task (Orr et al., 2008).

The sensorimotor region associated with the lower extremity exhibited a stronger correlation with SCALE than that associated with the upper extremity (Figure 2B). While activation of the sensorimotor cortex associated with the upper extremity would not be expected during ankle movement, the location of cortical activation has been found to vary for individuals with CP. Recently, motor evoked potentials with cortical stimulation were compared between adults with and without CP (Condliffe et al., 2019). Researchers reported that the “hotspots” for the soleus muscle of normal controls were in a tight cluster 2–3 cm lateral from the vertex (an anatomical landmark at the superior midpoint of the skull). For participants with CP, however, the hot spots were farther away from the vertex, more dispersed and, in some cases more lateral. Some hot spots appeared to be closer to the typical atlas site for the upper as compared to the lower extremity.

A unique aspect of this brain activation study was the inclusion of children with absent or very low levels of SVMC for whom greater cerebellar activation was found. Historically, the role of the cerebellum in normal movement production has been attributed to the control of balance and coordination. Although the exact role of the cerebellum is not fully known, studies have shown that it is involved with motor learning and motor control (Manto et al., 2012). Authors of a consensus article concluded that the cerebellar motor systems consist of an intrinsically connected network involved in the optimization of movement performance during the early phases of motor learning. It may also contain internal feedback models associated with unconscious skilled movement (Manto et al., 2012). This may explain increased activation in our participants with poor SVMC for whom this motor task was novel and more challenging.

An alternative explanation for greater cerebellar activation in children with reduced SVMC may be recruitment *via* alternative pathways that communicate with the cerebellum during movement. Although isolated ankle motion was requested, children with low SCALE scores produced simultaneous hip and knee motion (abnormal obligatory synergies). Rubrospinal tracts, which originate in the red nucleus and communicate with the cerebellum, have been associated with these more primitive synergies (Cahill-Rowley and Rose, 2014). Major afferents project from the cerebellar and cerebral cortices to the red nucleus and the rubrospinal tract projects to cerebellar nuclei before reaching the spinal cord (Darras and Volpe, 2018). Animal studies have shown that when the cerebellar nuclei are stimulated, stereotyped motor synergies are produced (Rispal-Padel et al., 1982). This may explain why children with larger deficits in SVMC may have a greater reliance on the cerebellum resulting in the production of less skillful patterned movements when higher centers of motor control are impaired.

While a stronger relationship with SCALE was found for the ipsilateral cerebellum, significance was also found for the contralateral side. Typically, there is an ipsilateral association between the cerebellum and limb movement; however, bilateral activation during unilateral movement has been reported in normal controls and adults post-stroke (Cui et al., 2000; Ehrsson et al., 2002; Nair et al., 2003; Kapreli et al., 2006; Dong et al., 2007). Normal adults performing a finger tapping task primarily activated their ipsilateral cerebellum when using their dominant hand but activated their cerebellum bilaterally when using their non-dominant hand (Dong et al., 2007). In the same study, patients post-stroke recruited their contralateral as well as their ipsilateral cerebellum while performing the task with their involved hand. While our patient population had bilateral rather than unilateral limb motor impairment, these data support our findings. Children with CP with greater impairment exhibited higher levels of bilateral cerebellar activation than those with less impairment.

### Limitations

This study had a small sample size but comparative fMRI studies contained even fewer participants with CP (Phillips et al., 2007; Hilderley et al., 2018). A control group of typically developing children was not studied. While the goal was to examine varying levels of brain metabolism based on selective motor control within CP, knowledge of normal activation patterns under the same conditions is unknown. A common limitation in PET is the low anatomical resolution when mapping metabolic activity. Further, there may be smoothing of the metabolic maps when they are resampled to the SPM anatomical template, especially due to the lack of subject-specific MRI anatomical scan. However, this smoothing may also potentially lead to a gain in the signal to noise ratio locally over the image. Finally, due to statistical correction for a large number of voxel-wise multiple comparisons, there may be a loss of power although this loss is mitigated by both cluster-wise thresholding methods and a separate *a priori* ROI-based analysis.

## Conclusion

In this study, we examined neuromotor control during an ankle motor task that was challenging for children with spastic CP, particularly for those with low levels of motor control. As we hypothesized, there was a significant positive relationship between SVMC and metabolic activity in the sensorimotor cortex that was contralateral to the moving limb. Interestingly, we found that lower motor control was associated with greater cerebellar activation during the motor task. Decreased cortical and increased cerebellar activation in children with more impaired motor control may be related to task difficulty, activation of new motor learning paradigms in the cerebellum and potential engagement of alternative motor systems when CSTs are focally damaged. These results support SCALE as a clinical correlate of neurologic damage.

## Data Availability Statement

All datasets generated for this study are included in the article.

## Ethics Statement

The studies involving human participants were reviewed and approved by Human Subjects Protection Committee, University of California, Los Angeles, USA. Written informed consent to participate in this study was provided by the participants’ legal guardian.

## Author Contributions

EF, WO, MG, and DS designed and conducted the experiments. EF, DS, and SJ analyzed the data. EF wrote the article. LS, SJ, and WO contributed to the discussion and edited the article.

## Conflict of Interest

The authors declare that the research was conducted in the absence of any commercial or financial relationships that could be construed as a potential conflict of interest.
